# Automatic respiratory and bulk patient motion corrected (ACROBATIC) free-running whole-heart five-dimensional magnetic resonance imaging

**DOI:** 10.1016/j.jocmr.2025.102673

**Published:** 2025-12-17

**Authors:** Robin Ferincz, Milan Prša, Estelle Tenisch, Jérôme Yerly, Christopher W. Roy

**Affiliations:** a Department of Radiology, Lausanne University Hospital (CHUV) and University of Lausanne (UNIL), Lausanne, Switzerland; b Division of Pediatric Cardiology, Woman, Mother-Child Department, Lausanne University Hospital (CHUV) and University of Lausanne (UNIL), Lausanne, Switzerland; c Center for Biomedical Imaging (CIBM), Lausanne, Switzerland

**Keywords:** 3D, Motion correction, Heart, Pediatric, Free-running, Radial

## Abstract

**Purpose:**

Free-running five-dimensional (5D) whole-heart magnetic resonance imaging (MRI) simplifies image acquisition by eliminating the need for external gating, breath-holding, and prospective scan planning. However, it remains vulnerable to patient movement in pediatric populations, which may require sedation or general anesthesia. We present a retrospective motion correction approach using the automatic respiratory and bulk patient motion correction (ACROBATIC) framework to detect, estimate, and correct for bulk motion, thereby improving image quality in pediatric cardiac MRI.

**Methods:**

Free-running Ferumoxytol-enhanced three-dimensional (3D) radial gradient-echo (GRE) data from 210 pediatric patients were manually categorized by the amount of bulk motion within each acquisition, based on retrospective reconstructions. From this cohort, 25 cases with the highest and 25 with the lowest detected bulk motion were selected, forming the moving and reference cohorts, respectively, for subsequent analysis and evaluation of the proposed framework. Respiratory motion was estimated using focused navigation. Bulk motion events were automatically detected from the variation in repeated radial readouts. The data were divided into four-dimensional (4D) arrays with timepoints spanning single respiratory cycles and reconstructed into retrospective real-time images using compressed sensing. Bulk motion was corrected via 3D rigid registration and poorly aligned images were excluded using an outlier-rejection algorithm. Final reconstruction was performed using a previously established 5D cardiac and respiratory motion-resolved compressed sensing approach. ACROBATIC’s performance was evaluated by a Dice coefficient (automatic detection), sharpness metrics at the blood-myocardium interface and within the pulmonary vessels, as well as qualitative grading by two expert reviewers.

**Results:**

The ACROBATIC framework successfully differentiated between moving and non-moving patients relative to manual evaluation (Dice = 0.96). Image sharpness significantly improved after motion correction, for analyses of the blood-myocardium interfaces and pulmonary veins. Expert evaluations supported the quantitative findings with average grade improvements of 0.44 and 0.54, respectively for Reviewer 1 and Reviewer 2.

**Conclusion:**

The ACROBATIC framework effectively reduces motion-related artifacts in pediatric cardiac MRI, particularly in patients with significant movement. This method supports the broader goal of achieving high-quality, dynamic whole-heart imaging in children without the need for sedation or general anesthesia.

## Graphical abstract

**Figure:**
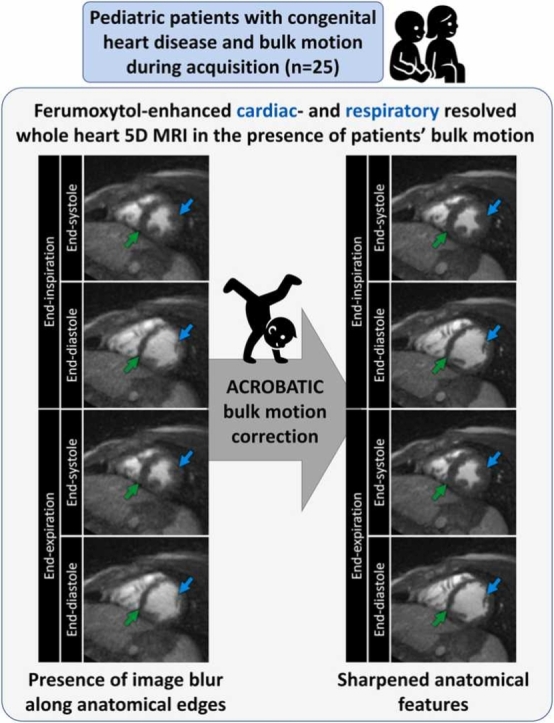

## 1. Introduction

Free-running cardiac and respiratory motion-resolved five-dimensional (5D) whole-heart magnetic resonance imaging (MRI) is increasingly used to assess cardiac anatomy and function [1], [2], [3], [4], [5], [6], [7]. Free-running acquisitions eliminate the need for external gating, triggering, and breath-holds through self-gating and motion-resolved reconstruction techniques. Compared to conventional two-dimensional (2D) cardiac magnetic resonance imaging (CMR), which has limited through-plane spatial resolution and requires precise scan planning, three-dimensional (3D) whole-heart MRI provides isotropic spatial resolution and improved patient comfort without the need for complex scan planning [8]. However, longer acquisition times make 3D whole-heart MRI more susceptible to motion artifacts [9], a common issue in pediatric patients, which may substantially degrade image quality.

One approach to address bulk motion is to repeat the scan when artifacts are detected. However, this may be unreliable, especially in pediatric populations, where motion is often involuntary and unpredictable. While sedation or general anesthesia can help reduce motion, these interventions carry risks such as hypoxemia [10] and should be used judiciously, especially for repeated imaging in children with congenital heart disease. An alternative strategy would be retrospective bulk motion correction [11], [12], [13], [14], [15], [16], [17], [18], [19], [20], [21]. While translational motion correction has been used for 3D whole-heart acquisitions [22] and rigid correction for 3D brain acquisitions [23], such methods have not yet been explored for 5D whole-heart MRI.

This study builds on the Automatic Respiratory And Bulk Patient Motion Correction (ACROBATIC) framework initially developed for 3D radial fetal MRI [21]. Our aim is to demonstrate the ability to correct bulk motion in 5D cardiac and respiratory motion-resolved reconstructions of radial whole-heart MRI acquisitions in pediatric patients with congenital heart disease. Here, we incorporate automatic detection of bulk motion, respiratory motion compensation through focused navigation [24], rigid registration of breath-to-breath reconstructions, and an outlier-rejection step to discard parts of the acquisition where bulk motion cannot be properly corrected. We hypothesize that the ACROBATIC framework can restore the diagnostic quality of motion-affected acquisitions to a level comparable to motion-free acquisitions.

## 2. Materials and methods

### 2.1. ACROBATIC framework

**Fig. 1:**
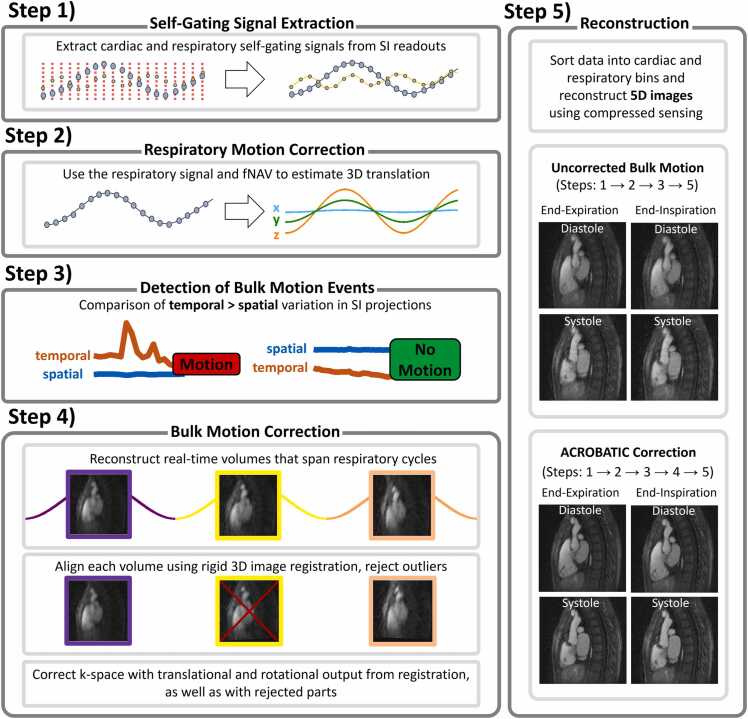
Schematic overview of the proposed ACROBATIC reconstruction framework. Step 1) Data are acquired during a free breathing free-running acquisition with a 3D radial spiral Phyllotaxis k-space trajectory with a radial SI readout at the beginning of each interleave to extract cardiac and respiratory self-gating signals. Step 2) Respiratory motion is estimated using the extracted respiratory signal and fNAV. Step 3) The detection of bulk motion events is performed by comparing the temporal variation of breath-to-breath SI projections to the respective spatial variation. Step 4) The data are divided into breath-to-breath intervals and reconstructed with compressed sensing. Each interval is aligned to an automatically chosen reference interval using 3D rigid registration. An outlier-rejection algorithm then discards parts of the acquisition that could not be properly aligned. The estimated motion and outlier-rejection information are then used to transform the original k-space data. Step 5) The k-space is then binned using the self-gating signals and reconstructed into 5D cardiac and respiratory motion-resolved images using compressed sensing with integrated motion fields. *ACROBATIC* automatic respiratory and bulk motion correction, *3D* three-dimensional, *SI* superior-inferior, *fNAV* focused navigation, *5D* five-dimensional.

The proposed ACROBATIC framework (Fig. 1) was designed for application to free-running 3D radial data with a spiral phyllotaxis k-space trajectory [25] and consists of five steps. First, cardiac and respiratory self-gating signals are extracted from the data [1]. Second, respiratory motion is estimated using focused navigation (fNAV) [24]. Third, bulk motion events are automatically detected. Fourth, bulk patient motion is estimated and corrected using image registration. Fifth, 5D images are reconstructed using compressed sensing with integrated cardiac and respiratory non-rigid motion fields and intra-bin respiratory motion correction [4], [5], [7]. The following sections provide a detailed description of each step in the proposed framework.

#### 2.1.1. Step 1: self-gating signal extraction

Extraction of cardiac and respiratory self-gating signals (Fig. 1) is performed as previously described [1] but briefly, the 3D radial spiral phyllotaxis k-space trajectory used in this work acquires a readout in the superior-inferior (SI) direction at the beginning of each spiral interleave. The Fourier transform of the SI readouts, hereafter referred to as SI projections, provides a 1D signal for each receiver coil channel and acquired time-point that includes the blood-myocardium and lung-liver interfaces. As such, these signals are modulated by the physiological motion that occurs during the acquisition [1]. The SI projections are processed using principal component analysis (PCA) and bandpass filtering, with frequency ranges tuned to the expected physiological motion: approximately 0.7–2.5 Hz and 0.1–0.7 Hz for cardiac and respiratory signals, respectively. These cardiac and respiratory self-gating signals will be used to bin our data for eventual 5D image reconstruction (step 5) but also to inform intra-bin motion compensation (steps 4&5) [4].

#### 2.1.2. Step 2: respiratory motion estimation

Displacement of the heart due to respiration is estimated using fNAV (Fig. 1) as previously described [24]. Briefly, the respiratory self-gating signal (step 1) is normalized and then scaled in each spatial direction (x, y, z) by three coefficients representing maximum displacement amplitudes. These initially unknown coefficients are iteratively estimated by minimizing the image gradient entropy [24] over a region of interest (ROI) in preliminary image reconstructions. The ROI is empirically selected as the central 64 voxels based on previous work [4], [24], [26]. These optimized coefficients can then be used for subsequent intra-bin compensation of respiratory motion (steps 4&5).

#### 2.1.3. Step 3: detection of bulk motion events

In practice, we may not know whether bulk motion has occurred during a scan. Therefore, to widely apply the ACROBATIC framework to free-running acquisitions but avoid erroneously correcting or rejecting data from patients that remained at rest, a method to automatically detect bulk motion (Fig. 1) was developed. We posit that the SI projections described above are also modulated by bulk patient movement. However, to decouple fluctuations in these signals due to bulk motion from cardiac and respiratory motion, the respiratory self-gating signal (step 1) can be used to identify the beginning and end of each respiratory cycle and then average together SI projections that belong to individual breathing cycles [21]. In this way, we create a set of modified SI projections as follows:

$$\tilde{\mathrm{SI}}(x,\mathrm{ch},t)$$

with signal from spatial coordinates (x), receiver coil channels (ch) and timepoints (t) representing individual breathing cycles. To detect bulk motion, consider the brightness constancy constraint from optical flow [27]:

$$\frac{\partial \tilde{\mathrm{SI}}(x,\mathrm{ch},t)}{\partial x}u+\frac{\partial \tilde{\mathrm{SI}}(x,\mathrm{ch},t)}{\partial t}=0$$

Which relates the spatial (∂/∂x) and temporal (∂/∂t) derivatives of $\tilde{\mathrm{SI}}$ (x, ch, t) via displacement vector (u). From this equation, we can show that:

$$\left|\frac{\partial \tilde{\mathrm{SI}}(x,\mathrm{ch},t)}{\partial x}\right|\left|u\right|=\left|\frac{\partial \tilde{\mathrm{SI}}(x,\mathrm{ch},t)}{\partial t}\right|$$

and therefore, if motion occurs (i.e., u>1 pixel per breathing cycle), the signal from the temporal gradient will exceed that from the spatial gradient. If we assume that displacement is approximately constant across our spatial coordinates, then we can average across each pixel (N_x_) to reduce sensitivity to noise. Furthermore, if we assume that bulk motion will be detected by each receiver channel (N_c_), we can also average across the coil dimension and simplify our equation to:

$${∆}_{s}(t)\left|u\right|={∆}_{t}(t)$$

where:

$${∆}_{s}\left(t\right)=\frac{1}{N_{c}}\frac{1}{N_{x}}\sum_{\mathrm{ch}=1}^{N_{c}}\sum_{x=1}^{N_{x}}\left|\frac{\partial \tilde{\mathrm{SI}}(x,\mathrm{ch},t)}{\partial x}\right|$$

and

$${∆}_{t}\left(t\right)=\frac{1}{N_{c}}\frac{1}{N_{x}}\sum_{\mathrm{ch}=1}^{N_{c}}\sum_{x=1}^{N_{x}}\left|\frac{\partial \tilde{\mathrm{SI}}(x,\mathrm{ch},t)}{\partial t}\right|$$

To account for bulk motion that occurs gradually over time, we evaluate ${∆}_{t}$ between every pair of timepoints (i.e., comparing every individual breathing cycle) and finally define bulk motion as having occurred if the following condition is met for at least one pair of timepoints as follows:

$${∆}_{s}(t)<{∆}_{t}(t)$$

If bulk motion has occurred, it is estimated and corrected (step 4), otherwise, 5D images can be reconstructed (step 5) by omitting bulk motion correction.

#### 2.1.4. Step 4: bulk motion correction

If bulk motion has been detected (step 3), it is estimated through the registration of 3D images (Fig. 1) that span individual respiratory cycles [21] as follows. Similar to the previous step, the respiratory self-gating signal is used to identify the beginning and end of each respiratory cycle, but rather than averaging SI projections, here we sort the acquired radial imaging lines to create bins that contain data from individual respiratory cycles giving us a four-dimensional 4D (x,y,z,t) k-space. To further decouple the impact of respiratory and bulk motion, we use the optimized fNAV coefficients (step 2) to correct the 4D k-space (intra-bin correction) by applying an appropriate phase shift. We then use compressed sensing with a spatial total variation regularization weight of 0.01 [28], [29] to reconstruct 4D images from which we can estimate bulk motion as follows. Bulk motion estimation is based on the registration of all timepoints from the 4D images to a reference. We calculate the reference image as a weighted average 3D image using a cost function based on mutual information as follows. Our 4D images (A) can be written as a flattened vector

$$A_{t}\in \;R^{x\cdot y\cdot z}$$

and then calculating the mutual information (MI) pairwise across all time points (T):

$$M(i,t)={\left[\mathrm{MI}\left(A_{i}A_{t}\right)\right]}_{i,t=1}^{T}$$

Taking the sum of the aggregated mutual information (M) then gives us a vector of weights (w_t_):

$$w_{t}=\sum_{i=1}^{T}M(i,t)$$

where for each timepoint (t), we have the mutual information between the image at that timepoint and all others. The weighted average 3D image is then given by:

$$A=\frac{1}{\sum_{t=1}^{T}w_{t}}\sum_{t=1}^{T}w_{t}\prime A_{t}$$

and represents an approximate median position of the subject throughout the acquisition. To estimate bulk motion, 3D rigid image registration is performed between the frames of the 4D image array and the reference 3D image over an ROI. As with fNAV (step 2), the ROI is empirically selected as the central 64 voxels based on previous work [21]. The cost function for image registration (mutual information) [30], [31] was minimized according to an evolutionary optimizer. After performing image registration, we assume that the 3D translational and 3D rotational output will allow us to correct the underlying bulk motion. Both 3D translations and 3D rotations are interpolated, using a Piecewise Cubic Hermite Interpolating Polynomial (PCHIP) [32] algorithm to match the timing of each spoke [15]. However, to account for motion that may not be well estimated or corrected by registration, we incorporate an outlier-rejection scheme as follows. We again calculate the mutual information weights (w_t_) described above but this time using the co-registered 4D images that are output from the 3D rigid image registration. Similarly, to the transformational output, the mutual information weights are interpolated to align with the timing of each spoke. We then fit a two-component Gaussian mixture model to the weights to identify outliers [33]. The two components are assumed to represent the well-aligned images (high mutual information values) and the not-well-aligned images (low mutual information values). Every spoke with an interpolated mutual information below the mean value of the lower mutual information Gaussian distribution is then considered as an outlier. Finally, the interpolated 3D translational and 3D rotational output from the rigid registration are then used to correct the acquired data through the application of an appropriate phase shift and rotation to the original k-space and trajectory coordinates, respectively. Additionally, any radial spoke corresponding to an identified outlier is rejected before final 5D image reconstruction. The entire bulk motion correction step is performed in a single iteration.

#### 2.1.5. Step 5: image reconstruction

Final reconstruction of 5D images utilizes the components of the previous steps (Fig. 1). The cardiac and respiratory self-gating signals (step 1) are used to sort the motion corrected k-space into 25 cardiac and 4 respiratory bins. Binning is performed on individual radial spokes independent of the original spiral interleave configuration. This combination of bins is based on previous work [4], [34] that has shown they provide adequate temporal resolution (∼20–50 ms depending on subject’s heart rate) to capture end-systole [35] while minimizing the impact of respiratory motion [36]. The impact of respiratory motion is further reduced using fNAV (step 2) to correct translational displacement within each bin (intra-bin correction) by applying an appropriate phase shift [4]. The binned k-space data (y) are reconstructed as cardiac and respiratory motion-resolved 5D image (m) in a two-part process. First, compressed sensing is performed as follows:

$$m=\underset{m}{\arg\min}{\left\|Fm-y\right\|}_{2}^{2}+\lambda_{r}{\left\|\nabla_{r}m\right\|}_{1}+\lambda_{c}{\left\|\nabla_{c}m\right\|}_{1}{+\lambda }_{s}{\left\|\nabla_{s}m\right\|}_{1}$$

with an operator (F) that contains coil sensitivities and the NUFFT, and with total variation-based regularization along the (∇_c_) cardiac (λ_c_ = 0.1), (∇_r_) respiratory (λ_r_ = 0.25) and (∇_s_) spatial (λ_s_ = 0.001) dimensions. From this first reconstruction, non-rigid registration is performed between adjacent cardiac and respiratory bins to create operators containing cardiac (T_c_) and respiratory (T_r_) deformation fields that can be used in a second final 5D image reconstruction ($\hat{m})$ where the impact of inter-bin motion blur caused by regularization is reduced [4], [5], [7]:

$$\hat{m}=\underset{m}{\arg\min}{\left\|\mathrm{Fm}-y\right\|}_{2}^{2}+\lambda_{r}{\left\|\nabla_{r}T_{r}m\right\|}_{1}+\lambda_{c}{\left\|\nabla_{c}T_{c}m\right\|}_{1}{+\lambda }_{s}{\left\|\nabla_{s}m\right\|}_{1}$$

### 2.2. Study data

To test the performance of ACROBATIC bulk motion event detection (step 3) and correction (step 4), we evaluated 210 pediatric patients with congenital heart disease who underwent MRI on a 1.5T clinical system (MAGNETOM Sola, Siemens Healthineers, Erlangen, Germany) after administering 2 mg/kg of Ferumoxytol (Covis Pharma Europe, Zug, Switzerland). A slab-selective, spoiled gradient echo free-running 3D radial sequence with spiral Phyllotaxis sampling was used, providing 6 minutes of uninterrupted acquisition time [4], [34]. Main sequence parameters were RF excitation angle: 15°, resolution: (1.15 mm)^3^, field of view: (220 mm)^3^, matrix size (192^3^), TE/TR: 1.53/2.84 ms, and readout bandwidth: 1002 Hz/pixel.

From this cohort, we manually identified subjects undergoing bulk motion to test the ACROBATIC framework. We retrospectively reconstructed 4D image arrays wherein each time point represented individual respiratory cycles as described in step 4. We then used an in-house graphical user interface (see additional file 1) that displayed orthogonal views and corresponding motion-mode (m-mode) images representing the temporal evolution of signal across interfaces within the images. Scrolling through each of the 210 subjects we (R.F.) graded the level of motion on a 5-point Likert scale with no perceived bulk movement (0), irregular respiration (1), one instance of minor bulk movement (2), multiple instances of bulk motion (3), and severe bulk motion (4). Examples of each motion grade are included as additional file 2. To test the impact of bulk motion correction we identified moving subjects by taking the 25 highest grades. However, in the absence of a non-moving reference for each subject we also created a cohort of 25 subjects with the lowest grades (no perceived bulk movement) for comparison. These three cohorts (all 210 patients, 25 moving, 25 reference) were used for the experiments described in the next sections.

### 2.3. Detection of bulk motion events

To evaluate the performance of our method for detecting bulk motion events, we performed step 3 of the reconstruction framework for all subjects (N = 210). We then compared the binary output of step 3 (bulk motion event detected/not detected) to the manually assigned grades described in the previous section and considering any grade greater than 0 as a motion event we calculated the Dice coefficient between the automatic and manual approaches.

### 2.4. Evaluating bulk motion estimation

To evaluate our approach for bulk motion estimation and correction, we performed step 4 of the reconstruction framework for all subjects from the manually identified moving cohort (N = 25). We recorded the 3D translational (x, y, z) components in mm and rotational (Rx, Ry, Rz) components in degrees from image registration wherein x, y, and z are the left-right, anterior-posterior, and superior-inferior directions, respectively. For a given subject, we recorded the average and peak motion. The amount of data rejected for each subject was also recorded to compare how much motion occurs during periods of the acquisition that are rejected.

### 2.5. Impact of bulk motion correction

To evaluate the overall performance of bulk motion correction (step 4), data from all subjects from the moving (N = 25) and reference (N = 25) cohorts were first reconstructed as 5D images, but omitting bulk motion correction and outlier rejection (uncorrected: steps 1,2,3&5). Then data from the moving cohort were reconstructed a second time using the full ACROBATIC framework including bulk motion correction and outlier rejection (corrected: steps 1–5). Note that the manually identified reference cohort was only reconstructed without bulk motion correction and outlier rejection (uncorrected) to provide a surrogate for an otherwise unavailable motion-free reference. 5D images were manually reformatted into short-axis and long-axis views. The motion estimates and resulting reconstructions were then quantitatively and qualitatively assessed as follows.

#### 2.5.1. Blood-myocardium interface sharpness analysis

The blood-myocardium interface sharpness was calculated as the sigmoidal slope in a 2D slice as previously described [37]. Perpendicular to the manually annotated line along the blood-myocardium interface, multiple sigmoidal functions (*n* = 100) were fitted to the corresponding signal intensity profiles. The interface sharpness was defined as the mean absolute slope of these fitted sigmoid curves (Fig. 5b) with a higher value indicating a sharper blood-myocardium interface. This process was repeated at the same anatomical locations for both uncorrected and corrected 5D images as described in the previous section.

#### 2.5.2. Pulmonary vein sharpness analysis

The pulmonary veins were used as an additional small feature “stress-test” of image sharpness. Soap-Bubble [38] was used to measure the vessel sharpness for the left and right pulmonary veins. Measurements were independently performed by two experts (R.F., C.W.R.), who traced the longest visible branch of the left and right pulmonary veins. This was performed for each uncorrected and corrected reconstruction in a blinded random order.

#### 2.5.3. Qualitative image grading

All reconstructed images were qualitatively evaluated with focus on the cardiac anatomy by two blinded expert reviewers (R1: M.P. pediatric cardiologist 13 years’ experience, R2: E.T. pediatric radiologist 12 years’ experience), using a 5-point Likert scale: non-diagnostic (0), marked blurring limited diagnostic value (1), moderate blurring diagnostic value (2), mild blurring good diagnostic value (3), excellent diagnostic quality (4). For each reviewer, the percentage of datasets rated as having diagnostic value or better (grades ≥2) was calculated. Additionally, the mean image quality grade and corresponding standard deviation (SD) were computed for each cohort. The average change in image quality grade from uncorrected to corrected was also determined for both reviewers across all datasets.

### 2.6. Statistics

Data distributions were assessed for normality using the Shapiro–Wilk test. A paired t-test was used to evaluate differences for normally distributed data; otherwise, the Wilcoxon signed-rank test or the rank sum test was applied, respectively for paired or unpaired data comparison. Statistical significance was set at p < 0.05.

### 2.7. Computation time

All reconstruction and evaluation steps were performed in MATLAB (MathWorks, Natick, Massachusetts), on a workstation equipped with a 24-core AMD Ryzen Threadripper PRO 7965WX CPU (AMD, Santa Clara, California), 1.0TB of RAM and a NVIDIA RTX 6000 Ada Generation GPU (Nvidia, Santa Clara, California). The computational time was recorded for individual steps of the reconstruction framework.

## 3. Results

### 3.1. Detection of bulk motion events

**Fig. 2:**
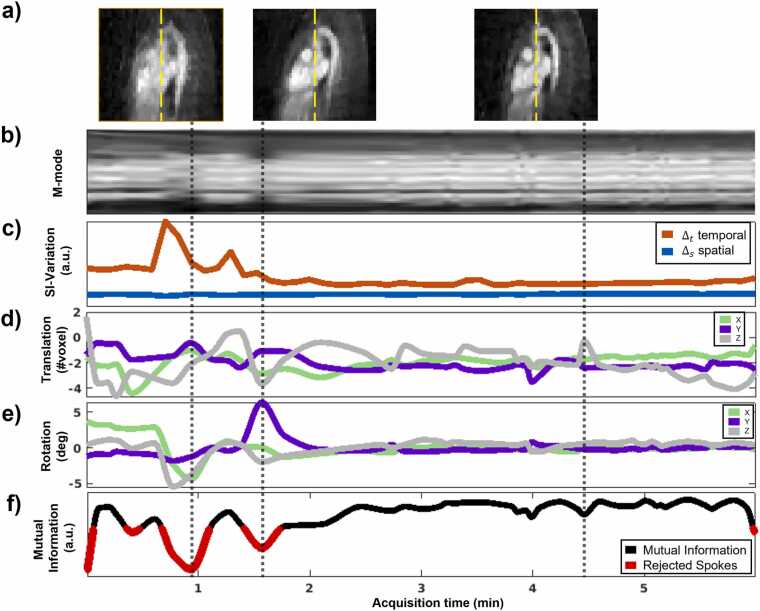
Visualization of the bulk motion assessment in the ACROBATIC framework. 4D images wherein each timepoint (a) corresponds to data averaged over individual respiratory cycles, enable the identification of the subject’s undergoing bulk motion with the temporal evolution of motion modes (m-modes) (b) (along yellow dashed lines). The spatial and temporal variation of SI projections (c), averaged over individual respiratory cycles, clearly identifies this subject as having multiple bulk motion events (∆_t_ > ∆_s_). Translational (d) and rotational outputs (e) from applying image registration to the 4D images (a) provide additional characterization and enable the correction of bulk motion. Outlier rejection employing a mutual information–based weighting function (f) discards data that cannot be aligned by the registration. *ACROBATIC* automatic respiratory and bulk patient motion correction, *4D* four-dimensional, *m-mode* motion mode, *SI* superior-inferior.

Overall, the proposed method for automatically identifying bulk motion events performed very well in the entire cohort (N = 210) when compared to manual assessment (Dice coefficient = 0.96). This approach is well visualized by an example subject with multiple bulk motion events (Fig. 2a, b). There were four subjects where no motion event was automatically detected, but had a manual grade of 1 (irregular breathing). Conversely, there were nine subjects where a motion event was falsely categorized by automatic detection but had a manual grade of 0 (no perceived bulk movement).

### 3.2. Evaluating bulk motion estimation

Translation and rotational motion components, reported as mean and standard deviation across all moving subjects (N = 25) showed low average displacement across the whole acquisition ([x: 0.58 ± 0.80, y: 0.55 ± 0.72, z: 0.83 ± 0.96] mm, [Rx: 0.47 ± 0.56, Ry: 0.59 ± 0.70, Rz: 0.45 ± 0.53] °) but with peak values that correspond well with bulk motion events ([x: 3.33 ± 2.88, y: 2.27 ± 1.32, z: 4.04 ± 2.28] mm, [Rx: 2.30 ± 1.85, Ry: 3.40 ± 2.02, Rz: 2.49 ± 1.82] °). The mean and standard deviation of rejected data as a percentage of acquisition length was 9.3% ± 6.1% across all moving subjects. The average displacement during periods of the acquisitions identified as outliers were higher than during the full acquisition ([x: 1.50 ± 1.93, y: 0.82 ± 0.94, z: 1.59 ± 1.77] mm, [Rx: 0.77 ± 0.85, Ry: 1.44 ± 1.48, Rz: 0.87 ± 0.87] °) but with similar peak values ([x: 3.29 ± 1.93, y: 2.08 ± 1.31, z: 3.85 ± 2.39] mm, [Rx: 1.92 ± 1.12, Ry: 3.24 ± 2.07, Rz: 2.21 ± 1.27] °) suggesting that outliers are largely identified as motion events that exceeded the tolerance of the image registration. This was confirmed by an example subject (Fig. 2), where the rejected regions (Fig. 2f), correspond to parts in the acquisition with high estimated translational (Fig. 2d) and rotational (Fig. 2e) values.

### 3.3. Impact of motion correction on image quality

**Fig. 3:**
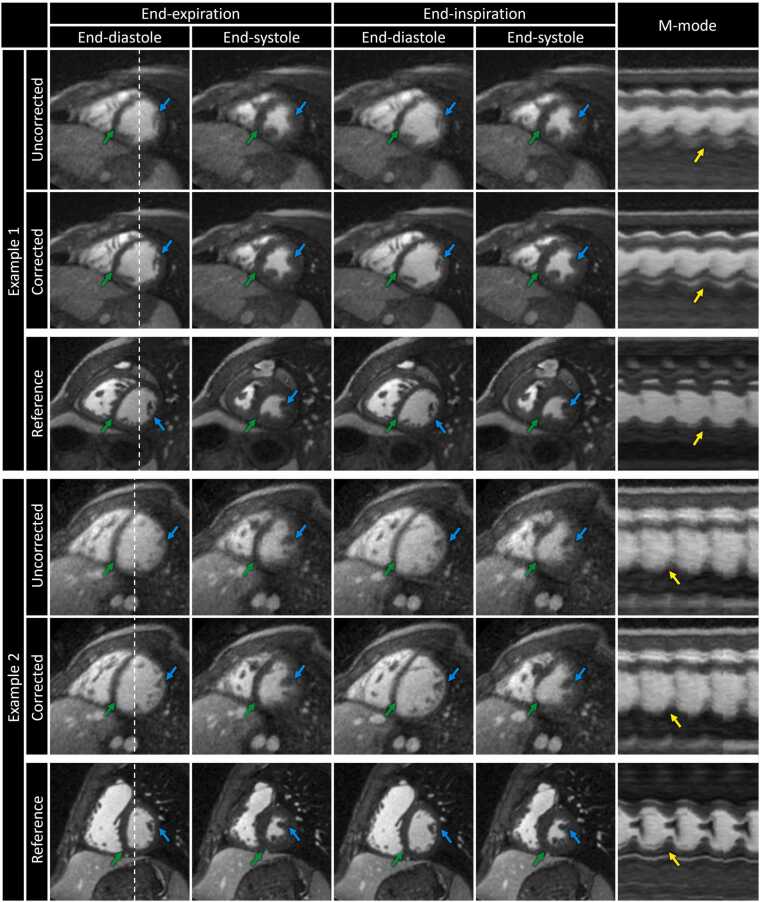
Reconstructions of exemplary subjects in short-axis views during end-diastole and end-systole for end-expiratory and end-inspiratory respiratory phases. The temporal evolution of signals throughout the cardiac and respiratory cycles along the dashed line is displayed as an m-mode image. Uncorrected and corrected 5D images are shown for two moving subjects along with reference images from separate non-moving subjects. Fine details that improve with motion correction include the blood-myocardium interface (green arrows), papillary muscles (blue arrows), and blood-myocardium interface at end-systole (yellow arrows). Animated versions of the reconstructions shown here are included in Additional file 3. *M-mode* motion mode, *5D* five-dimensional.

Uncorrected 5D images from moving subjects demonstrated noticeable blurring of the blood-myocardium interface (green arrows) and papillary muscles (blue arrows) in short-axis views during end-diastole, end-systole, end-expiration and end-inspiration (Fig. 3). Additionally, contraction of the heart throughout the cardiac and respiratory cycles appeared more blurred when considering the temporal evolution of signal (yellow arrows) bisecting the left ventricle (Fig. 3: m-mode). Conversely, applying ACROBATIC correction yielded visibly sharper features that approach the quality of those found in 5D images from non-moving reference subjects.

**Fig. 4:**
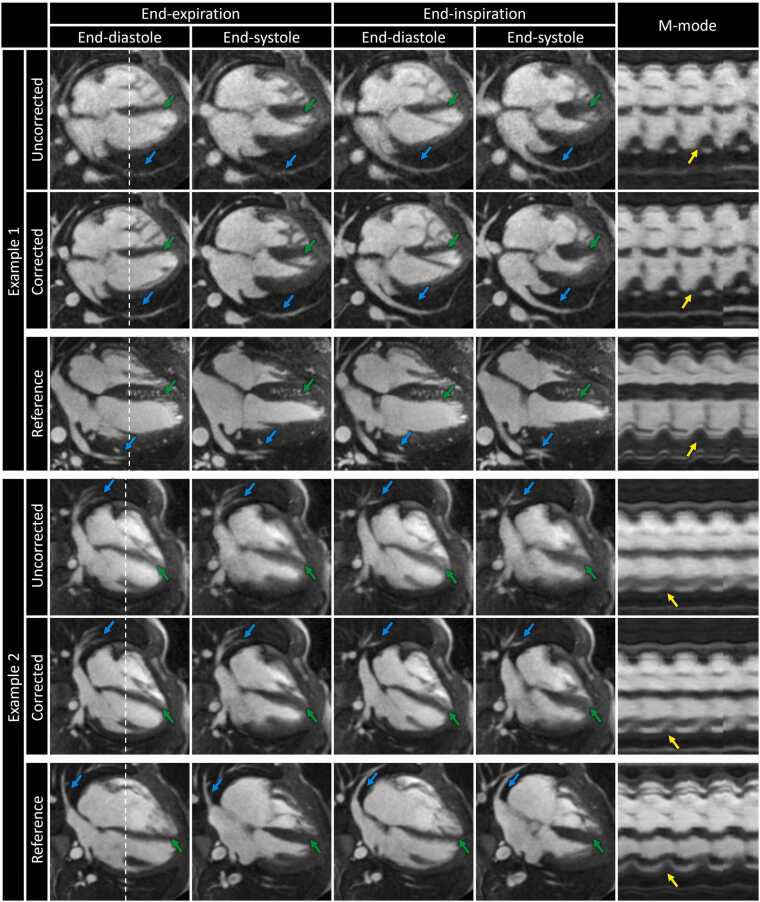
Reconstructions of exemplary subjects in long-axis views during end-diastole and end-systole for end-expiratory and end-inspiratory respiratory phases. The temporal evolution of signals throughout the cardiac and respiratory cycles along the dashed line is displayed as an m-mode image. Uncorrected and corrected 5D images are shown for two moving subjects along with reference images from separate non-moving subjects. Fine details that improve with motion correction include the blood-myocardium interface (green arrows), pulmonary veins (blue arrows), and blood-myocardium interface at end-systole (yellow arrows). Animated versions of the reconstructions shown here are included in Additional file 3. *M-mode* motion mode, *5D* five-dimensional.

Similarly, uncorrected 5D images from moving subjects resulted in noticeable blurring of the blood-myocardium interface (green arrows), pulmonary veins (blue arrows), and cardiac contraction (yellow arrows) in long-axis views (Fig. 4). Again, ACROBATIC correction resulted in better definition of all fine structures with nearly comparable quality to 5D images from reference subjects.

#### 3.3.1. Blood-myocardium interface sharpness analysis

**Fig. 5:**
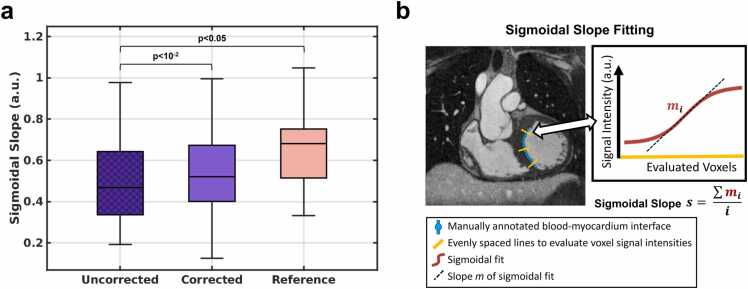
Blood-myocardium interface sharpness. Uncorrected 5D images are compared to corrected images from 25 moving subjects in addition to 25 reference images from non-moving subjects. Bars indicate statistically significant difference, determined with a Wilcoxon signed rank test (and ranked sum test for unpaired comparison with the reference data). b) Visualization of the sigmoidal slope fitting with the manually annotated blood-myocardium interface in a coronal 2D slice of the heart on the left ventricle and evenly spaced lines to evaluate voxel signal intensities (left), sigmoidal fitting, and the calculation of the sigmoidal slope (right). *5D* five-dimensional, *2D* two-dimensional.

Blood-myocardium interface sharpness (Fig. 5) reported as median values with interquartile ranges [Q1–Q3], varied for uncorrected (0.47 [0.34–0.64]), corrected (0.52 [0.40–0.67]), and reference 5D images (0.68 [0.51–0.75]). However, while uncorrected images were significantly less sharp than both corrected (p < 10^−2^) and reference (p < 0.05) images, there was no significant difference between corrected and reference images (p = 0.08), reflecting the image quality shown in Fig. 3, Fig. 4.

#### 3.3.2. Pulmonary vein sharpness analysis

**Fig. 6:**
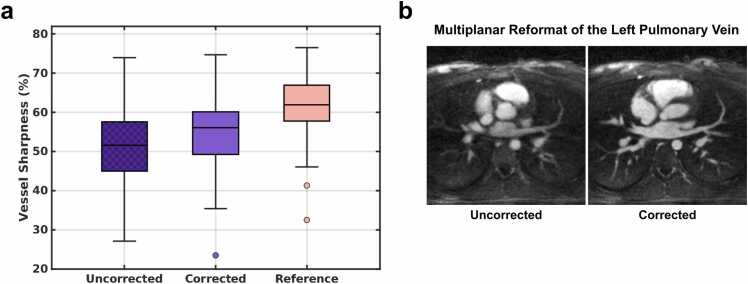
a) Pulmonary vessel sharpness. Uncorrected 5D images are compared to corrected images from 25 moving subjects in addition to 25 reference images from non-moving subjects. All three datasets (uncorrected, corrected, reference) are showing statistically significant difference towards each other, determined with a Wilcoxon signed rank test (and ranked sum test for unpaired comparison with the reference data). b) Exemplary multi-planar reformat of the left pulmonary vein for one uncorrected and one ACROBATIC corrected reconstruction of the same moving subject. *5D* five-dimensional, *ACROBATIC* automatic respiratory and bulk patient motion correction.

Pulmonary vessel sharpness analysis (Fig. 6a) reported as median values with interquartile ranges [Q1–Q3], also varied between uncorrected (51.57 [44.96–57.53] %), corrected (56.05 [49.24–60.10] %), and reference (61.93 [57.76–66.88] %) 5D images. Corrected (p < 10^−3^) and reference (p < 10^−3^) images had significantly sharper vessels than uncorrected images, but here there was also a significant difference between corrected and reference images (p < 10^−3^). An exemplary 2D reformat of a left pulmonary vein from a moving subject underlines these findings with visually sharper vessels in corrected 5D images (Fig. 6b).

#### 3.3.3. Qualitative image grading

**Fig. 7:**
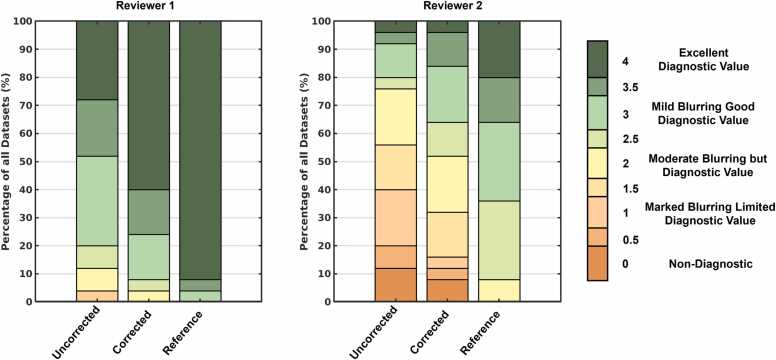
Image grading from two expert reviewers. Uncorrected 5D images are compared to corrected images from 25 moving subjects in addition to 25 reference images from non-moving subjects based on a 5-point Likert scale.

Expert image grades (Fig. 7) reported as mean and standard deviation also varied. Both reviewers reported lower grades for uncorrected images (R1: 3.18 ± 0.76, R2: 1.64 ± 1.09), than for corrected (R1: 3.62 ± 0.56, R2: 2.18 ± 1.08) images albeit with lower overall grades from R2. However, despite an average half-grade improvement after applying correction (R1: +0.44, R2: +0.54), the reference images still had the highest grades (R1: 3.94 ± 0.22, R2: 3.06 ± 0.63). Nevertheless, ACROBATIC correction led to an increase in images with diagnostic value or better (R1: 96% to 100%, R2: 48% to 68%) with 100% of the reference images achieving diagnostic value or better.

### 3.4. Computation time

The total computational time for applying the ACROBATIC framework to one dataset was approximately 6 h for all steps. Self-gating signal extraction (step 1): ∼1 min. Respiratory motion estimation using fNAV (step 2): ∼2 min. Automatic detection of bulk motion events (step 3): ∼1 min. Compressed sensing reconstruction of 4D arrays for bulk motion estimation (step 4): ∼ 50 min. Image registration for bulk motion estimation (step 4): ∼8 min. Final 5D image reconstruction using compressed sensing and motion fields (step 5): ∼5 h.

## 4. Discussion

In this study, the ACROBATIC framework, originally developed for fetal imaging [21] was adapted and applied to free-running radial whole-heart MRI acquisitions in pediatric patients with congenital heart disease. A comprehensive analysis showed that the ACROBATIC framework enabled the automatic detection, estimation, and correction of bulk patient motion yielding cardiac and respiratory motion-resolved 5D images in non-compliant patients. We successfully tested our hypothesis that ACROBATIC 5D image reconstructions have improved image quality in moving subjects relative to the previously established uncorrected 5D method with results that approach the quality of reference images from patients without noticeable bulk motion.

### 4.1. Detection of bulk motion events

The proposed method for automatically detecting bulk motion events (step 3) was based on principles from optical flow applied to the repeated SI projection that is part of the 3D radial spiral Phyllotaxis k-space trajectory used in this work. This approach provided robust identification of datasets wherein the patient moved and excellent agreement with manual assessment across 210 datasets (Dice: 0.96). Automatically classifying data plays an important role in applying the ACROBATIC framework widely to datasets without a priori knowledge about patient compliance. If erroneously applied to patients at rest, bulk motion estimation from image registration and outlier rejection becomes sensitive to noise rather than motion which may lead to slight degradation of image quality. On the other hand, if data contains motion but is falsely classified, images may be seriously blurred. In this way, the current approach, which correctly identified all but four cases of minor motion, provides a useful foundation for the remaining steps of the framework. Nevertheless, a key component of this approach involved decoupling the effects of respiratory and bulk motion by averaging SI projections within individual respiratory cycles. As a result, there remains some sensitivity to irregular breathing that may be picked up as bulk motion and potentially corrected or rejected.

### 4.2. Bulk motion correction and outlier rejection

Bulk motion estimation and correction was based on image registration applied to 4D image arrays wherein each timepoint represented one respiratory cycle. Overall, motion correction clearly led to improved image quality suggesting bulk motion was well tracked and corrected. However, in the absence of ground truth estimates of the underling motion, further validation is required to establish the boundaries of the ACROBATIC framework for 5D imaging. This may be achieved using numerical phantoms [21]. Nevertheless, as with our bulk motion detection approach, the need to decouple the effects of respiratory and bulk motion may result in intra-bin motion blur of the individual images we use for registration. This may lead to inaccurate estimations of motion as well as erroneous data rejection due to the relatively long temporal footprint for each image (5–10 s depending on respiratory rate). Enhancing the temporal resolution could allow us to estimate respiratory and bulk motion at the same time resulting in more accurate motion correction and overall improved image quality. Strategies to achieve higher temporal resolution may include the use of different trajectories with more efficient k-space coverage per unit time, such as other radial approaches [39], [40] or spiral cones trajectories [41], as well as the incorporation of advanced reconstruction techniques for the sub-volumes, including low-rank models [42] or sliding-window methods [43]. Additional approaches to enhance 3D bulk motion estimation could involve transitioning to non-rigid bulk motion models [44], which are capable of capturing more realistic and non-uniform motion patterns across the assessed ROI.

Outlier rejection was based on a Gaussian mixture model and the assumption that even after image registration, some data may remain unaligned due to the motion being greater than can be tolerated by the registration algorithm or because the 4D image array contains images with too much blur due to intra-bin motion. This effectively divides the co-registered data into two states, a corrected and an uncorrected one. We therefore fit two Gaussian distributions to our mutual information weighing function (see step 4) to automatically classify these two states and reject data from the distribution with the lowest values. As a result, approximately 10% of the acquired radial data were rejected on average for moving subjects. Outlier rejection may be improved by exploring additional similarity metrics other than mutual information or by assessing other statistical methods to classify the data such as thresholds on the mean or median values. Nevertheless, outlier data corresponded largely to timepoints where the largest translational and rotation motion was measured, supporting the current approach.

### 4.3. Impact of motion correction on image quality

ACROBATIC 5D image reconstructions, including automatic detection, estimation, correction, and rejection of bulk motion resulted in significantly higher quantitative measures of sharpness (blood-myocardium interface, pulmonary veins) and expert grading of image quality relative to previously established uncorrected 5D imaging. However, while image quality after bulk motion correction approached that of reference images, residual uncorrected motion, or rejection of on average 10% of the acquired data may still lead to some degradation relative to uncorrected, inherently motion-free acquisitions. As such, improvements to bulk motion estimation and outlier rejection may still be needed to bridge the gap. Furthermore, expert reviewers arrived at different baselines for assessing subjectively defined diagnostic value in the moving subjects but generally agreed when assessing the reference images. This may further point to residual variability in image quality even after correction. Nevertheless, both consistently found an improvement in image quality when applying the ACROBATIC correction that increased the number of subjects with diagnostic value or better.

## 5. Limitations

Image reconstructions were performed offline, with a computation time of approximately 6 h per dataset. Most of this time was attributed to the final compressed sensing reconstruction incorporating motion fields, required to generate 5D cardiac and respiratory motion-resolved images [4], [5], [7]. This processing time inherently impacts the clinical translation of this approach, however, advances in artificial intelligence are showing promise to replace or accelerate the reconstruction process [45]. Such advances may enable the integration of the ACROBATIC framework directly within scanner hardware through emerging third-party reconstruction interfaces [46], [47], [48].

In this study, the pediatric cohort was injected with Ferumoxytol as a contrast agent prior to the MRI exam as part of the clinical protocol at our institution, which led to an increased blood-myocardium contrast due to the T1-shortening effect. Nevertheless, all individual components of the ACROBATIC framework, including fNAV, rigid motion correction, outlier rejection, and compressed sensing, have been previously included in studies with native contrast [1], [7], [21]. Still, the overall performance of ACROBATIC for different contrast regimes needs to be assessed as the current approach certainly benefits from the high blood signal provided by Ferumoxytol to track cardiac, respiratory, and bulk motion. If successful in other contrast regimes, this may help minimize the risks associated with contrast agent use in pediatric populations.

Beyond pediatric MRI, the ACROBATIC framework demonstrates broad translational potential, including applicability in neonatal and adult cohorts, and even non-cardiac assessment for motion robust cerebral [49] or abdominal [50] whole organ imaging. Its extension to flow-sensitive acquisitions and integration with spectrally selective fat suppression may further support hemodynamic assessment [51] and coronary imaging [9], [52], [53], respectively, aligning with the goal of enabling 5D diagnostics of cardiac anatomy with volumetric whole-heart coverage robust to bulk patient motion.

## 6. Conclusion

The ACROBATIC framework enabled Ferumoxytol-enhanced pediatric 5D whole-heart MRI despite the presence of bulk patient motion. Through effective suppression of motion artifacts, the ACROBATIC framework shows strong potential to enhance image quality in non-compliant patients potentially reducing the need for re-scanning or sedation, particularly important for pediatric patients who require lifelong follow-up care.

## Funding

CWR is the principal investigator on 10.13039/501100001711Swiss National Science Foundation Grant PZ00P3_202140 that funded this research.

## Author contributions

**Robin Ferincz:** Writing – review & editing, Writing – original draft, Visualization, Validation, Methodology, Investigation, Formal analysis, Data curation, Conceptualization. **Prsa Milan Prša:** Writing – review & editing, Writing – original draft, Validation, Formal analysis, Data curation. **Estelle Tenisch:** Writing – review & editing, Writing – original draft, Validation, Formal analysis, Data curation. **Jérôme Yerly:** Writing – review & editing, Writing – original draft, Methodology, Formal analysis, Data curation. **Christopher W. Roy:** Writing – review & editing, Writing – original draft, Visualization, Validation, Supervision, Project administration, Methodology, Investigation, Formal analysis, Data curation, Conceptualization.

## Ethics approval and consent

All subjects or their legal guardian in the case of minors provided informed written consent, including permission to publish anonymized data, as part of a study approved by the local ethics review board (CER-VD 2022–01521).

## Declaration of competing interests

The authors declare the following financial interests/personal relationships which may be considered as potential competing interests: The authors receive non-monetary research support from Siemens Healthineers.

## Data Availability

The datasets and algorithms used and analyzed during the current study are available from the corresponding author upon reasonable request.
