# Supplementary material for: Automatic respiratory and bulk patient motion corrected (ACROBATIC) free-running whole-heart five-dimensional magnetic resonance imaging
Source: J Cardiovasc Magn Reson. 2025 Dec 17;28(1):102673. doi: 10.1016/j.jocmr.2025.102673 (PMC12814851; doi:10.1016/j.jocmr.2025.102673)
Supplement: Supplementary file 1 — Supplementary material [file mmc1.docx]

# Supplementary Material:


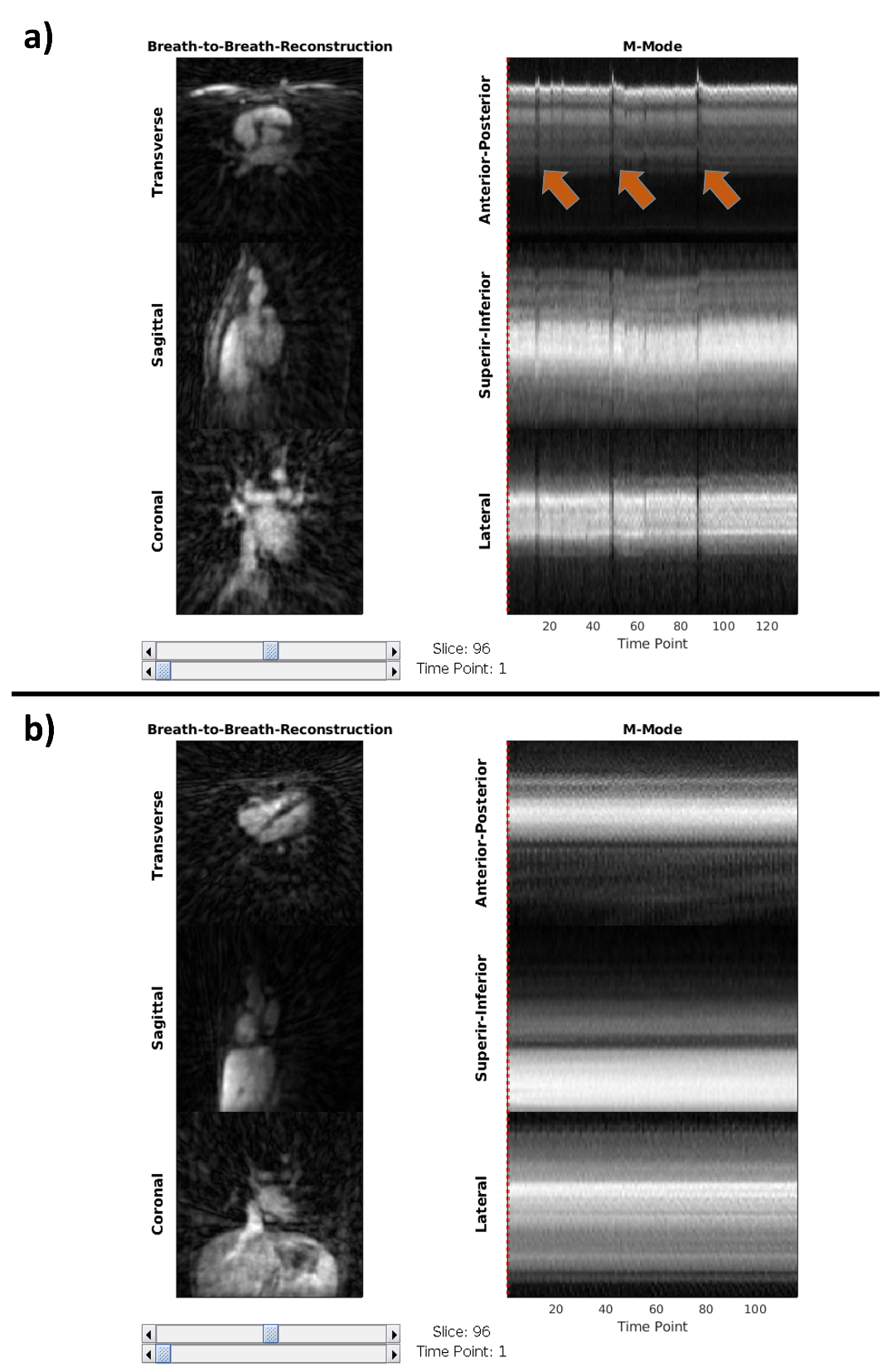


*Additional File 1*: Graphical user interface for classification of MR data based on bulk motion characteristics. The interface enables visual assessment and categorization of motion artifacts across MR acquisitions. Left panel: Three orthogonal views (transverse, sagittal, coronal) of a representative under-sampled breath-to-breath reconstruction. Right panel: Corresponding m-mode representations in anterior-posterior, superior-inferior, and lateral directions. A red-dotted line denotes the temporal location of the reconstruction shown in the left panel. Two interactive sliders (bottom left) allow adjustment of the time point and slice position for dynamic visualization. (a) Dataset exhibiting pronounced bulk motion, with three distinct motion events indicated by orange arrows in the m-mode plots. (b) Dataset with minimal motion, showing no notable bulk motion throughout the acquisition. *M-mode, motion-mode.* *This figure should be printed in color.*


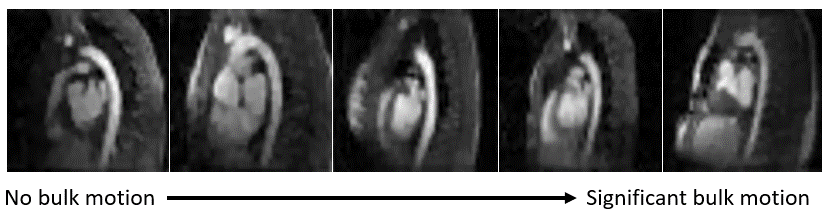


*Additional file 2:* Examples of dynamic breath-to-breath reconstructions from 5 of the initial 210 pediatric patients in sagittal view to illustrate different levels of bulk patient motion occurring throughout the data acquisition.


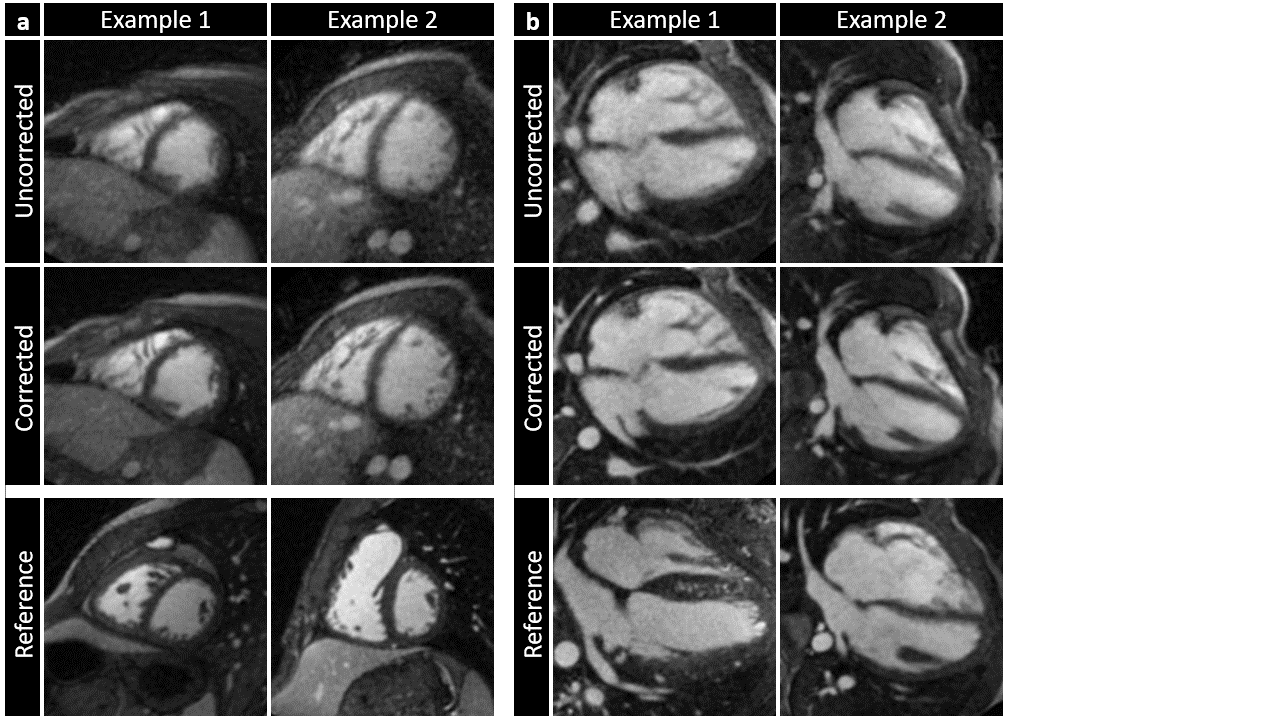


*Additional file 3*: Dynamic cardiac resolved reconstructions of exemplary subjects from Figure 3 (a) and Figure 4 (b) at end-expiration in a) short-axis and b) long-axis views throughout the cardiac and respiratory cycle.
